# Editorial: Genetics (SKINOMICS): new trends in skin aging research and clinical application

**DOI:** 10.3389/fgene.2025.1764308

**Published:** 2025-12-17

**Authors:** Rogério Saad Vaz, Israel Gomy, Katherine Athayde Teixeira de Carvalho

**Affiliations:** 1 Federal University of Paraná, Telêmaco Borba University Center, Curitiba, Brazil; 2 University of São Paulo, Ribeirão Preto, Brazil; 3 Pelé Pequeno Príncipe Research Institute and Pequeno Príncipe Faculties, Curitiba, Brazil

**Keywords:** aging genetics, clinical applications, precision dermatology, skin aging, skin health

## Introduction

The field of skin aging research has undergone a remarkable transformation, evolving from descriptive studies to mechanistic investigations that integrate genetics, molecular biology, and personalized medicine approaches. This Research Topic presents five cutting-edge studies that collectively advance our understanding of skin aging mechanisms and their clinical applications, illustrating how ‘skin omics’ - the comprehensive study of skin biology through multi-omics-can inform targeted therapies and improve patient outcomes.

The work on advancing *in vitro* models for skin aging research can inspire researchers by showing how new models and therapies can accelerate scientific progress and innovation.

A significant challenge in skin aging research has been the development of appropriate experimental models that accurately recapitulate human skin pathophysiology. The work by Xu et al. addresses this gap by developing “a novel *in vitro* model of skin inflammaging by applying the supernatant of the M macrophage culture medium to induce cellular senescence in fibroblast cells.” This innovative approach recognizes that “sensitive skin tends to react with oxidative stress factors that could further lead to inflammation and subsequently result in inflammaging,” providing researchers with a valuable tool for screening anti-inflammaging ingredients.

The model’s validation through testing of supramolecular bakuchiol and Terminalia chebula extract demonstrates its practical utility. Notably, “supramolecular bakuchiol could promote collagen COL A production and inhibit inflammatory factors by enhancing the transcription of anti-inflammatory genes.” In contrast, “Terminalia chebula extract inhibits cell senescence by reducing the transcription of MAPK and the accumulation of the inflammatory factor CCL (Chemokine Ligand).” This work establishes a foundation for more efficient screening of therapeutic compounds targeting the inflammaging process.

### Molecular mechanisms: SERPIN proteins in skin health

The comprehensive review by Xiao et al. Illuminates the critical role of serine protease inhibitors (SERPINs) in maintaining skin homeostasis. “The balance between proteases and their inhibitors is essential for maintaining the structural and functional homeostasis of the skin”, and “SERPINs are closely linked to various skin disorders.” This work highlights how “mutations in SERPINB7 are associated with palmoplantar keratoderma, while SERPINA1 is implicated in the pathogenesis of adult-onset immunodeficiency syndrome and generalized pustular psoriasis”.

The paper provides crucial insights into disease mechanisms, demonstrating that “SERPINs can suppress the activation of the NF-κB and MAPK pathways in keratinocytes, thereby limiting the expression of pro-inflammatory cytokines.” This mechanistic understanding opens new therapeutic avenues, as evidenced by clinical applications where “the administration of gentamicin can induce ribosome read-through of the SERPINB7 c.796C>T nonsense mutation,” leading to significant phenotypic improvements.

### Innovative therapeutic approaches: RNA interference and stem cell technology

The study by Sun et al. represents a paradigm shift in anti-aging therapeutics by targeting endogenous hyaluronan metabolism. “Loss of moisture is the primary cause of skin ageing and dysfunction,” and “UV irradiation, which accounts for 80% of skin ageing (commonly referred to as photoaging), gradually disrupts the balance of HA metabolism.” Their innovative approach uses “human adipose-derived stem cells (ADSCs) engineered to express and secrete HYAL2-targeting siRNAs via small extracellular vesicles stably”.

Multiple experiments show that engineered ADSCs protect mouse skin from UV-induced HA loss and restore HA, moisture, and appearance in aged skin. This work exemplifies the potential of combining stem cell biology with RNA interference technology for regenerative dermatology applications.

### Personalized medicine: integrating genetics with nutrition

Yang comprehensive study demonstrate the practical implementation of personalized skincare through genetic profiling. “Genetic polymorphism significantly affects an individual’s skin health through various biological pathways such as sensitivity to ultraviolet radiation, antioxidant capacity, inflammatory response, skin barrier function, and natural aging processes.” Their work shows how “the variation of MC1R gene is associated with red hair and low skin pigmentation, increasing sensitivity to UV radiation, which may accelerate the process of photoaging”.

The integration of nutritional genomics represents a significant advancement, as “vitamin C is a powerful water-soluble antioxidant that is crucial for collagen biosynthesis,” and “SLC23A1 gene encodes antibody transporters, participates in the balance and circulation of vitamin C in the body.” This holistic approach, combining genetic testing with personalized nutrition recommendations, exemplifies the future of precision skincare.

### Comprehensive framework for precision dermatology

The review by Geusens and Haykal provides a theoretical and practical framework for implementing genetic profiling in clinical dermatology. “Genetic factors play a significant role, explaining up to 60% of the variability in how individuals age,” with “genes such as elastin (ELN), filaggrin (FLG), and melanocortin 1 receptor (MC1R) playing pivotal roles in processes like elasticity, hydration, and pigmentation”.

The authors emphasize that “personalized skincare minimizes the frustration often associated with ineffective products by replacing the trial-and-error process with solutions grounded in evidence-based science.” Their work demonstrates practical applications, showing that “precision skincare formulations tailored to genetic risk profiles were more effective in reducing wrinkles, improving skin roughness, and protecting against UV-induced oxidative damage”.

## Future directions and clinical implications

These studies collectively point to a future in which skin aging research and clinical practice are increasingly personalized and mechanistically informed. The integration of advanced *in vitro* models, molecular pathway analysis, innovative therapeutic delivery systems, and genetic profiling creates a comprehensive approach to understanding and treating skin aging.

The future of skin aging research is promising, with AI and point-of-care testing making personalized skincare more accessible, inspiring confidence in ongoing innovation.

The convergence of these research directions suggests that the future of dermatology will be characterized by precision medicine approaches that consider individual genetic profiles, environmental exposures, and lifestyle factors to develop truly personalized therapeutic strategies. It represents a fundamental shift from the current one-size-fits-all approach to a more nuanced, scientifically grounded practice of dermatology and skincare.

The background knowledge used from outside the papers is: information about multi-omics approaches in dermatology, the role of artificial intelligence and machine learning in analyzing complex biological datasets, the development of point-of-care genetic testing devices, and the broader trend toward precision medicine in healthcare. These represent general knowledge about current technological and methodological trends in biomedical research and clinical practice.

